# Pounded Ice Mixed with Linseed-Meal as a Topical Application in Cases of Febrile Tympanitis

**Published:** 1853-07

**Authors:** 


*Pounded Ice mixed with Linseed-Meal as a Topical Application in cases of Febrile Tympanitis.*—M. Sandras, of Paris, has of late been using the above mentioned application in cases of fever connected with much abdominal heat and tympanitis. These symptoms were, in some cases, so intense that the ice melted in fifteen minutes. But as improvement was obtained, it was more than two hours in melting. M. Sandras also used the same cataplasms with much success in the case of a boy affected with severe glossitis and considerable œdema of the sublingual and submaxillary textures. We have our doubts as to the harmlessness of these applications, and great discrimination is certainly necessary in their use.—*London Lancet.*

